# Irreducible Intertrochanteric Fractures: Analysis of Various Fracture Patterns and Reduction Techniques

**DOI:** 10.7759/cureus.75014

**Published:** 2024-12-03

**Authors:** Anupam Gupta, Dinakar Rai

**Affiliations:** 1 Orthopaedics, PSG Institute of Medical Sciences and Research, Coimbatore, IND; 2 Trauma and Orthopaedics, PSG Institute of Medical Sciences and Research, Coimbatore, IND

**Keywords:** ao classification, epilson sign, femoral irreducible intertrochanteric fractures, irreducible intertrochanteric fractures, lessor trochanter, pfna-2 implant

## Abstract

Background

Numerous classifications exist for intertrochanteric (IT) fractures, commonly focused on stability. However, the currently utilized Arbeitsgemeinschaft Osteosynthesefragen and Orthopaedic Trauma Association (AO/OTA) classification has limitations in identifying irreducible fractures. This study aims to answer the following questions: does fracture stability imply irreducibility; which fracture fragments complicate reduction; and which reduction techniques should be employed?

Materials and methods

Eligibility criteria included fractures in adult long bones without pathological fractures being treated by native conservative means. Preoperative pelvic X-rays were obtained from 49 patients who had intertrochanteric fractures and classified according to the 2018 AO Classification. Anterior-posterior pelvic X-rays were reviewed by six experienced surgeons, who reached a consensus on type, group, and subgroup classifications in this prospective observational study. The methods for intraoperative fracture reduction used by five different unit chiefs were recorded and tabulated. All fractures were reduced on a fracture table with traction and rotation and subsequently checked under C-arm imaging. Persistent non-anatomical alignment with displacement was classified as an irreducible IT fracture. Various reduction techniques, using either semi-open or open methods, were analyzed.

Results

Fractures classified as AO types A1.1, A1.3, and A2.1 were generally more reducible, while types A2.2, A2.3, A3.1, A3.2, and A3.3 were more frequently irreducible.

Patients under 65 years of age were more likely to present with irreducible fracture patterns (P = 0.026), a statistically significant association. A semi-open method using spikes or Hohman’s retractors was most commonly employed, with no preliminary cortical fixation using K-wires after reduction.

Conclusion

Irreducible fractures exhibit unique features on C-arm imaging, potentially leading to increased anxiety and longer operation times. Awareness of these fracture characteristics can assist surgeons in achieving effective reduction and reducing operation time. The 2018 AO classification alone does not reliably predict irreducible IT fractures.

## Introduction

The reason why irreducibility prediction is clinically significant is that it helps young surgeons to be mentally prepared to do either semi-open methods of reduction or open reduction and internal fixation. Thereby reducing radiation and operative time. The Arbeitsgemeinschaft Osteosynthesefragen (AO) classification, an alphanumeric system, designates specific bones and fracture sites. In this system, the femur is identified as ‘3,’ with the proximal segment as ‘1’ and the trochanteric site labeled as ‘A.’ However, this classification has limitations in identifying irreducible intertrochanteric (IT) fractures.

Irreducible fractures are those where traction and rotation on a fracture table fail to achieve anatomical alignment under C-arm imaging [1,2]. This study aims to identify irreducible IT fractures and to analyze various patterns and techniques adopted for reduction. This randomized prospective observational analysis intends to address new research questions: are unstable IT fractures always irreducible; can irreducibility be predicted by an anterior-posterior (AP) X-ray of the pelvis; and which reduction techniques should be employed?

Previous studies have identified irreducible fractures, yet variables in fracture patterns remain unpredictable [3-6]. Determining which fractures are likely to be irreducible can be challenging, as the AO classification relies on plain AP X-rays [7]. This prospective study reports on (A) classified IT fractures, both reducible and irreducible, as well as techniques adopted for cortical contact and anatomical alignment.

## Materials and methods

Since proximal femoral nail anti-rotation is a standardized surgery, information from lateral X-rays and CT scans does not change the surgical outcomes. The patients are exposed to unnecessary radiation and costs. In general, the classification should be based on traction and rotation on a fracture table with C-arm imaging to define irreducible fractures.

This prospective randomized study was conducted at PSG Institute of Medical Sciences & Research, Coimbatore, and received approval from the Institutional Human Ethics Committee of the teaching institution (PSG/IHEC/2023/Qry/035/EXP, dated 20/02/23), with informed consent obtained in writing from all participants.

The prospective study included 49 patients who underwent proximal femoral nail anti-rotation (PFNA2) fixation for IT fractures between October 2022 and October 2023. The average age of the participants was 67 years, and all underwent standard plain X-rays of the pelvis at the time of injury. No lateral view X-rays or CT scans were performed. Patients with preoperative traction applied were included in the study, while those with delayed presentations or who received native treatment were excluded. Demographics and fractures were classified using the 2018 AO classification and recorded in a Microsoft Excel (Microsoft Corporation, Redmond, Washington, United States) spreadsheet.

Each patient was anesthetized and positioned supine on a fracture table, undergoing traction and rotation for reduction under C-arm imaging. Two attempts were made to assess the fracture patterns: traction and internal rotation, and traction and neutral rotation. Fractures that could not be reduced by traction and rotation were categorized as irreducible, and images were documented. Patients were operated on by five different unit chiefs within 36 hours postinjury, with reducibility evaluated using Kims et al. reduction criteria without modification [8]. In brief, the proximal head and neck fragment were positioned within the cortical thickness of the distal fragment, as observed in two C-arm imaging views.

Reduction techniques for irreducible fractures, as performed by the five chiefs, were documented in a spreadsheet. The acceptable reduction criteria included continuity of the medial and anterior cortex, as well as the absence of sag. In other words, the head and neck fragments were required to remain within the cortical bone thickness.

Statistical analysis was conducted on the AO types of fractures, correlating reducibility with irreducibility, and the techniques for reduction were tabulated.

## Results

Patients with AO types A1.1, A1.2, and A2.1 were stable and were reducible on the fracture table, while those with A2.2, A2.3, A3.2, and A3.3 were more likely to be unstable and be irreducible on the fracture table on C-arm imaging (Table 1).

**Table 1:** Cross tabulation of AO types on traction and rotation. AO: Arbeitsgemeinschaft Osteosynthesefragen

						Chi-square value	P-value
		Irreducible		Reducible			
		Count	Row N %	Count	Row N %		
AO classification	A1.1	0	0.0%	1	100.0%	18.826	0.016
	A1.2	4	30.8%	9	69.2%		
	A1.3	4	66.7%	2	33.3%		
	A2.1	0	0.0%	6	100.0%		
	A2.2	6	85.7%	1	14.3%		
	A2.3	7	77.8%	2	22.2%		
	A3.1	2	50.0%	2	50.0%		
	A3.2	1	100.0%	0	0.0%		
	A3.3	2	100.0%	0	0.0%		

With different fracture patterns, the chi-square value is 18.826, and the P-value is 0.016.

The surgical techniques adopted for AO types showed no significant and specific pattern of fracture to be irreducible; in other words, some AO types and subgroup types of fractures cannot be predicted in all ways for irreducibility (P-value = 0.467) (Table 2).

**Table 2:** The surgical techniques for AO types showed not much significant association with reduction criteria. AO: Arbeitsgemeinschaft Osteosynthesefragen

Cross-tabulation of AO classification and surgical techniques		Surgical techniques										P-value
		Anterior Spikes/Elevator Count		Open		Orif clamp		Semi-open		Spikes		
		Count	Row N %	Count	Row N %	Count	Row N %	Count	Row N %	Count	Row N %	
AO classification	A1.2	0	0.0%	0	0.0%	0	0.0%	3	75.0%	1	25.0%	0.467
	A1.3	0	0.0%	2	66.7%	0	0.0%	1	33.3%	0	0.0%	
	A2.2	0	0.0%	0	0.0%	0	0.0%	3	50.0%	3	50.0%	
	A2.3	1	14.3%	1	14.3%	2	28.6%	2	28.6%	1	14.3%	
	A3.1	0	0.0%	0	0.0%	1	50.0%	1	50.0%	0	0.0%	
	A3.2	0	0.0%	0	0.0%	0	0.0%	0	0.0%	1	100.0%	
	A3.3	0	0.0%	1	50.0%	0	0.0%	0	0.0%	1	50.0%	

Patients under 65 years of age sustained irreducible fractures, which were statistically significant; the chi-square value for age was 4.978, and the P-value was 0.026 (Table 3).

**Table 3:** Patients under 65 years of age were more likely to have an irreducible fracture, while those over 65 were more likely to have a reducible fracture. There was a statistically significant association between age group and fracture status (p = 0.026) AO: Arbeitsgemeinschaft Osteosynthesefragen

Patient on fracture table with traction and rotation for AO types fracture		Irreducible		Reducible			P-value
		Count	Column N %	Count	Column N %	Chi-square value	
Age	<65	15	57.7%	6	26.1%	4.978	P value=0.026
	>66	11	42.3%	17	73.9%		
Gender	F	15	57.7%	12	52.2%	0.150	P value=0.698
	M	11	42.3%	11	47.8%		
AO classification	A1.1	0	0.0%	1	4.3%	18.826	P value=0.016
	A1.2	4	15.4%	9	39.1%		
	A1.3	4	15.4%	2	8.7%		
	A2.1	0	0.0%	6	26.1%		
	A2.2	6	23.1%	1	4.3%		
	A2.3	7	26.9%	2	8.7%		
	A3.1	2	7.7%	2	8.7%		
	A3.2	1	3.8%	0	0.0%		
	A3.3	2	7.7%	0	0.0%		

No association of gender with irreducible fractures specifically noted, with a chi-square value of 0.150 and a P-value of 0.698.

According to the 2018 AO classification, we found a positive chi-square value of 18.826 and a P-value of 0.016 for fractures of different types. It meant subgroup types of classification are more prone to irreducible on the fracture table yet cannot be accurately predicted since some types of fracture fragments reduce by traction and rotation on the fracture table.

The semi-open surgical technique was commonly practiced by surgeons, while open reduction was done with a clamp in only 12% of cases. The mean, standard deviation, frequency, and percentage were taken on record. Variables were compared using Pearson’s chi-square test, with a significance level of P < 0.05 in a two-tailed test, and analysis was conducted using IBM SPSS Statistics for Windows, Version 21 (Released 2012; IBM Corp., Armonk, New York, United States).

About 53.1% are being irreducible, largely due to more cases under 65 years, and 46.9% were reducible fractures (Table 4).

**Table 4:** There was no statistically significant association between AO classification and sex (p = 0.698). AO: Arbeitsgemeinschaft Osteosynthesefragen

Distribution of intertrochanteric fractures on traction and rotation		Count	Column N %
AO classification	A1.1	1	2.0%
	A1.2	13	26.5%
	A1.3	6	12.2%
	A2.1	6	12.2%
	A2.2	7	14.3%
	A2.3	9	18.4%
	A3.1	4	8.2%
	A3.2	1	2.0%
	A3.3	2	4.1%
Reducible	Irreducible	26	53.1%
	Reducible	23	46.9%
Surgical techniques	Anteriorly spikes/Elevator	1	4.0%
	Open	4	16.0%
	Orif clamp	3	12.0%
	Semi open	10	40.0%
	Spikes	7	28.0%
Age	<65	21	42.9%
	>66	28	57.1%
Gender	F	27	55.1%
	M	22	44.9%
Side	Left	26	53.1%
	Right	23	46.9%

## Discussion

The anatomy of the trochanteric region of the femur is described to understand fractures, which are variable. The large quadrangular bony projection seen with continuity with the neck and shaft is the trochanter.

Medially there is a trochanteric fossa, and the lateral surface blends distally with the femoral shaft. A small conical projection at the posterior inferior aspect of its junction with the neck is the lesser trochanter.

A vertical plate called calcar femorale ascends from the compact bone near the linea aspera into the trabeculae of the neck. The intertrochanteric line is a prominent ridge at the junction of the anterior surfaces of the neck and shaft. Upper and lower bands of the iliofemoral ligament are attached [9].

All classifications for trochanteric fractures are based on the factor of stability. Despite adequate fixation, fractures that collapse before union are considered unstable, and this occurrence can occur with any fixation device. The AO classification has divided fractures into three groups: posterior medial comminution fractures, lesser trochanter fractures, and lateral wall fractures. However, this classification is not suitable for stating whether the fracture is reducible or irreducible.

This study analyzed which fractures can be irreducible, with no diagnostic CT scan imaging preoperatively, and assessment was purely based on a plain X-ray of the pelvis and intraoperatively by C-arm imaging.

The quality of intraoperative reduction under traction and rotation under C-arm imaging clearly demonstrated an irreducible fracture fragment in non-anatomical alignment. Closed reduction has been ineffective in approximately 17.6% of the cases [8,10].

However, in our study, 53.1% of the fractures were irreducible due to 21 patients younger than 65 years sustaining the fracture due to high-velocity trauma.

**Figure 1:**
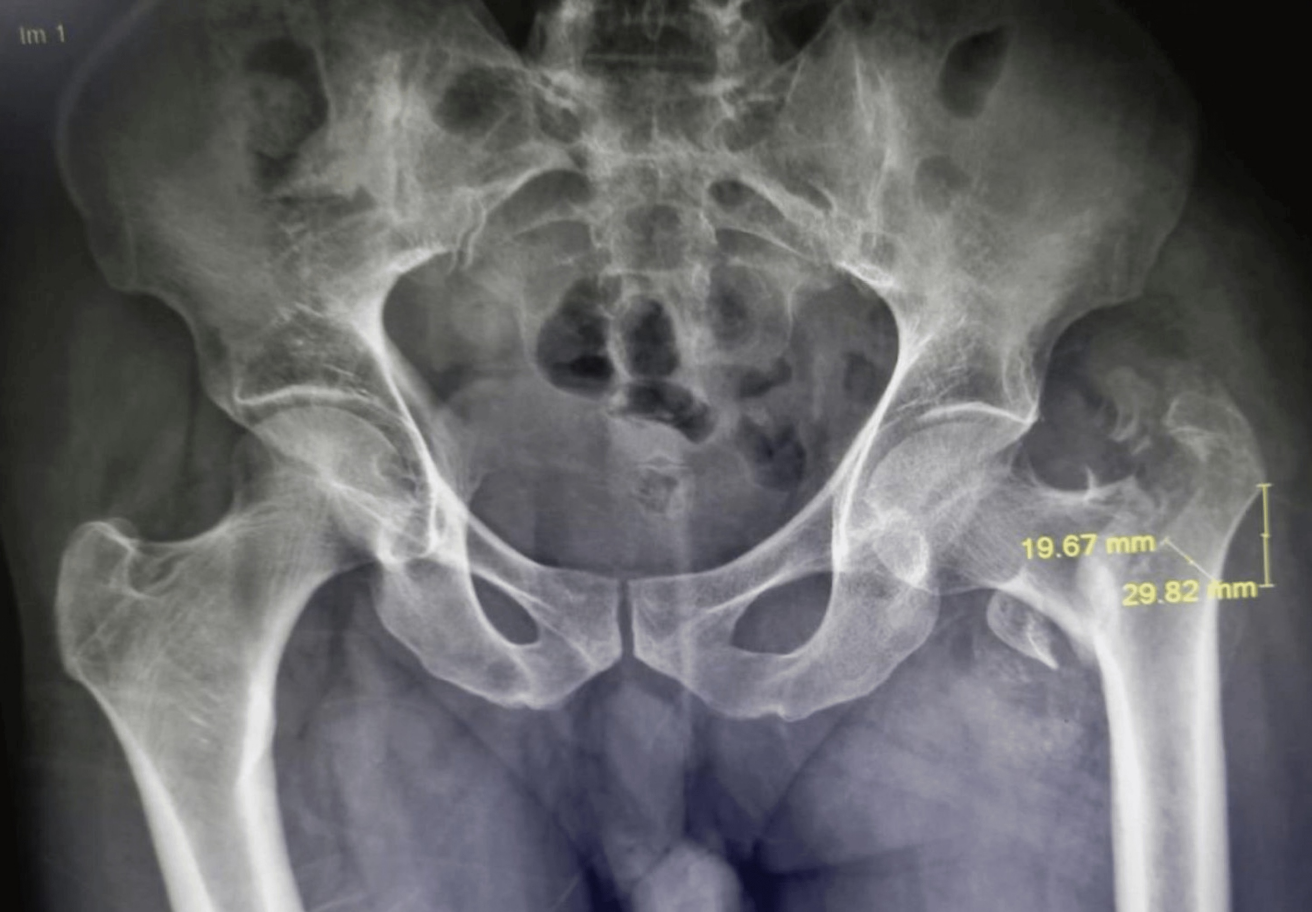
Lesser trochanter detached closer to the neck (irreducible fracture intertrochanteric). Lateral wall intact type AO/OTA 31a1.3 AO/OTA: Arbeitsgemeinschaft Osteosynthesefragen and Orthopaedic Trauma Association

Ikutta et al. [11] reported the combined use of lateral view X-ray and CT scan images with Jensen’s classification for prediction of fracture irreducibility and stated that lesser trochanters moving more proximal towards the neck of the femur were irreducible, which was also found in our study (Figure 1).

Said et al. [12] found that when the distal fragment included the lesser trochanter, it got buttonholed between the iliopsoas tendon and the lesser trochanter, presenting a variant and needing surgical release for reduction. In our study we found impaction of fragments, which required open reduction.

Hao et al. [13] reported that 67% of reverse oblique fractures were irreducible and identified three predictors: displacement of the shaft medially required open reduction with clamp, displacement of the lesser trochanter, and broken and displaced lateral wall. In our study, how the broken lateral wall made the fracture irreducible is unclear since only plain X-rays were taken.

**Figure 2:**
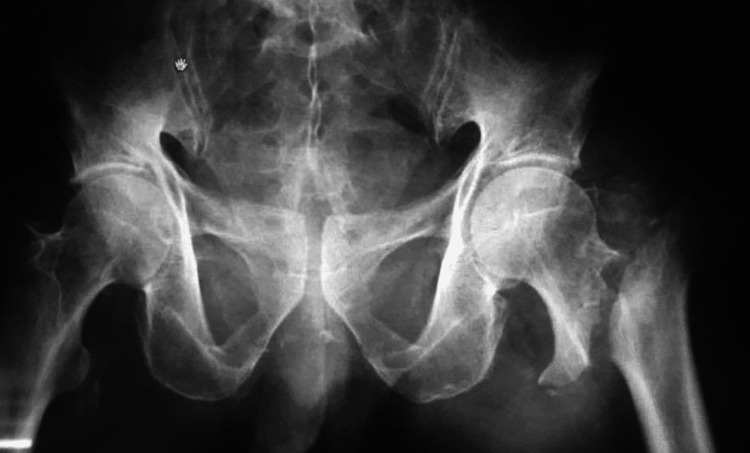
Epsilon sign on the left side with long spike head and neck fragment, predicts irreducibility of the fracture. [17]

Raj et al. [14] mentioned CT scans imaging enabled them to be reclassified as A2.2-A3.3 from A1.1 and A2.1; these were irreducible types that are consistent with our study [15,16]. In our study, the finding of the “Epsilion sign,” as a predictor, was not encountered as stated by Chandak et al. [17]. This sign appears as a “3” and reverse “3”- Epsilon sign on plan X-ray of the pelvis (Figure 2).

**Figure 3:**
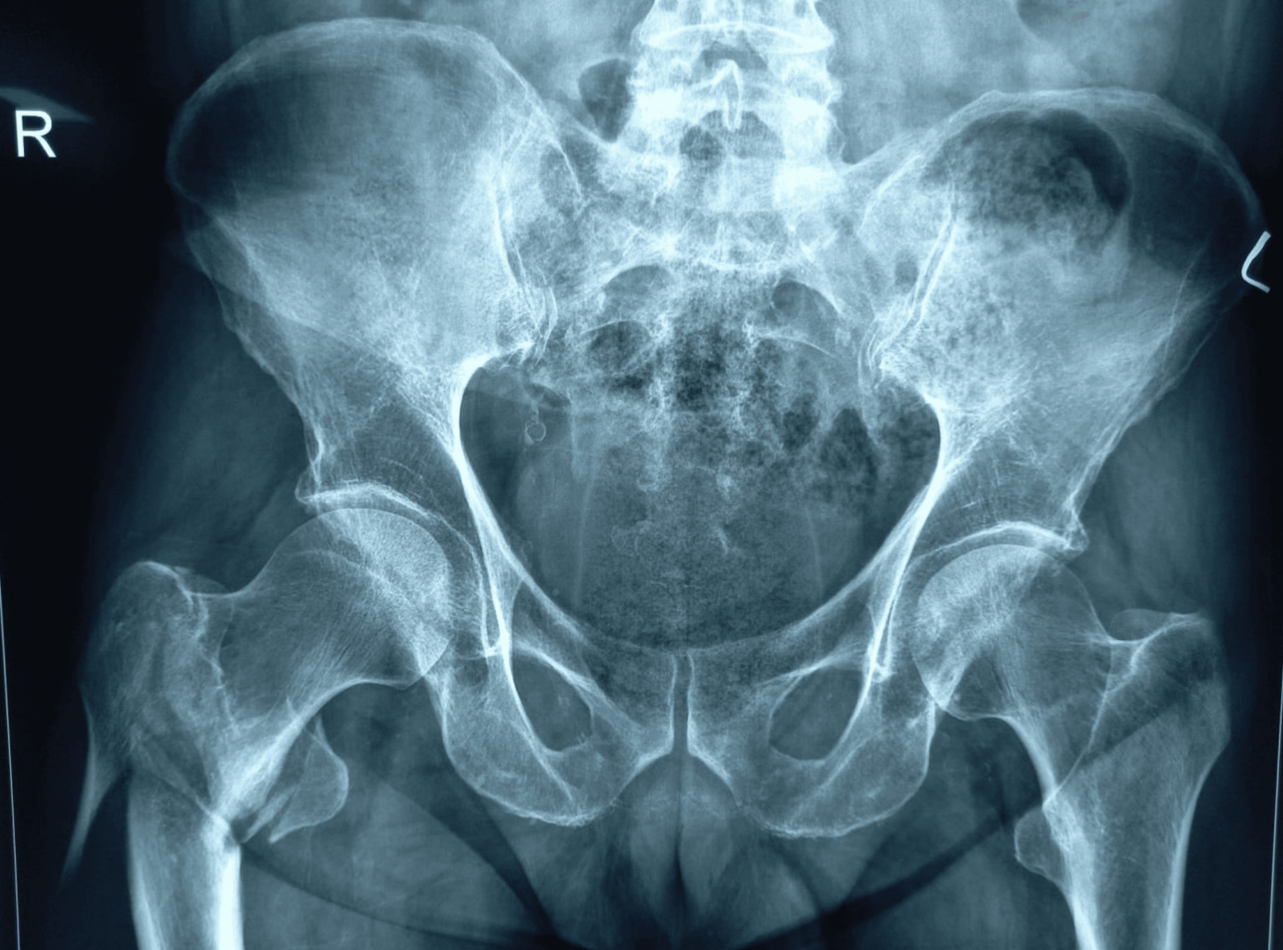
Fracture shows detached lesser trochanter, fracture of lateral wall, distal fragment between lateral wall and intermediate fragment of head and neck but no varus. (AO 31a3.3) AO: Arbeitsgemeinschaft Osteosynthesefragen

**Figure 4:**
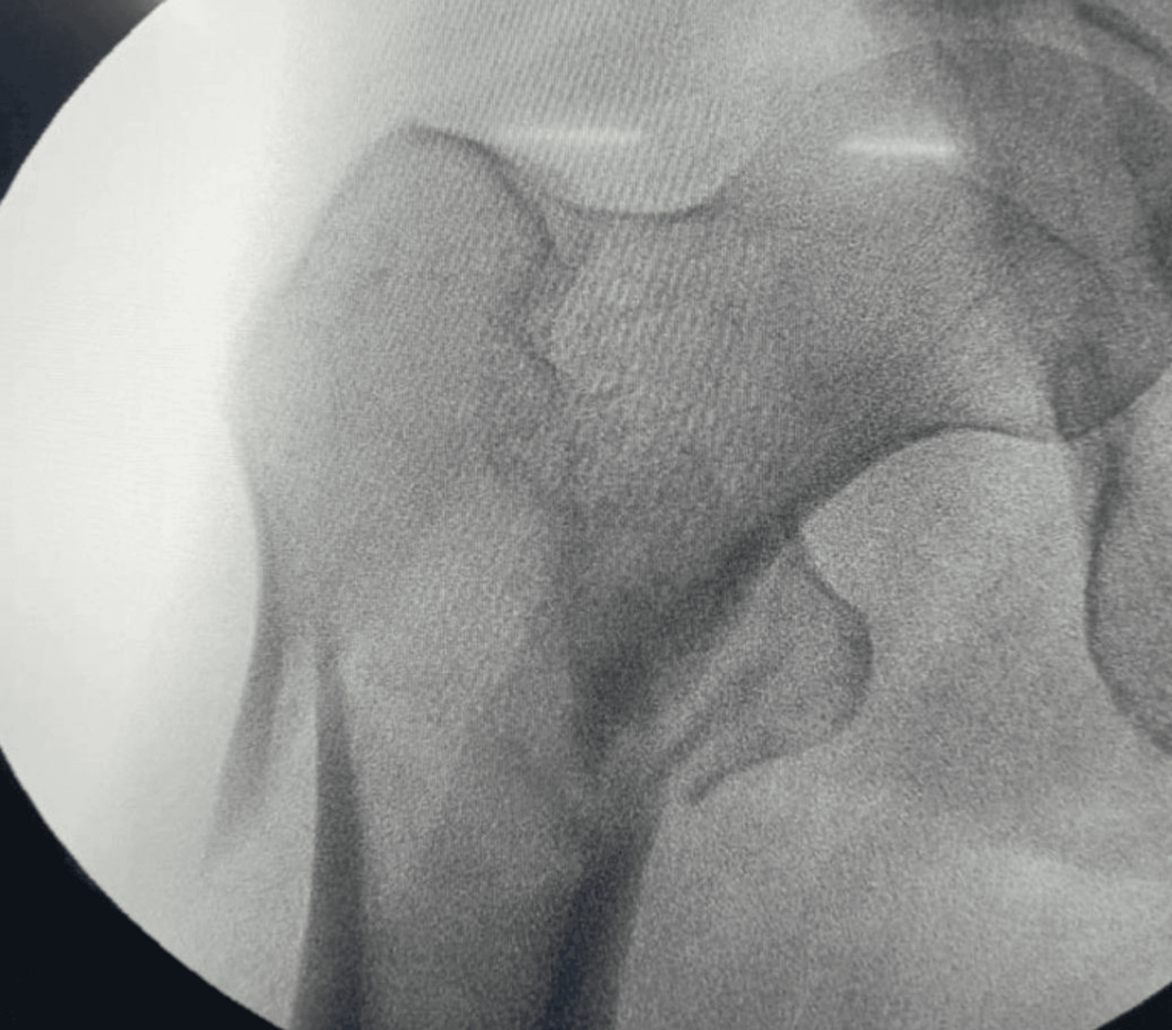
C-arm view shows anatomical alignment in anterior-posterior view.

**Figure 5:**
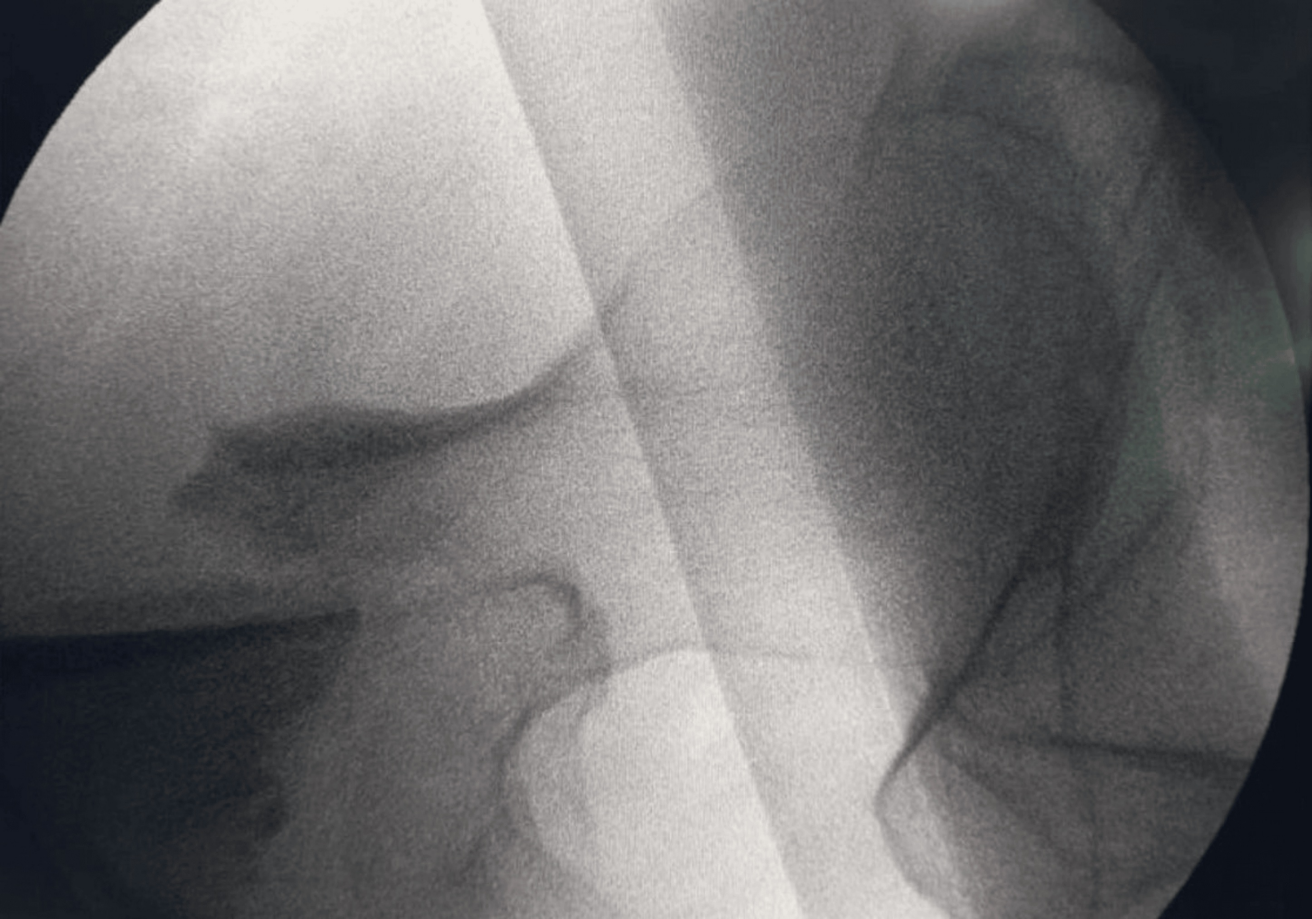
C-arm image on lateral view shows head and neck anteriorly displaced.

**Figure 6:**
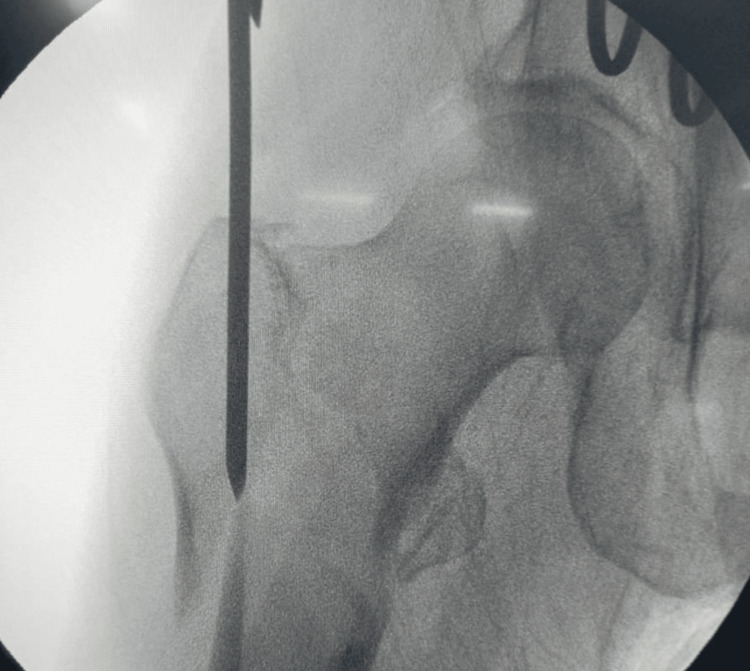
On an AP view, guide wire and alignment of head and neck with shaft in valgus. AP: anterior-posterior

**Figure 7:**
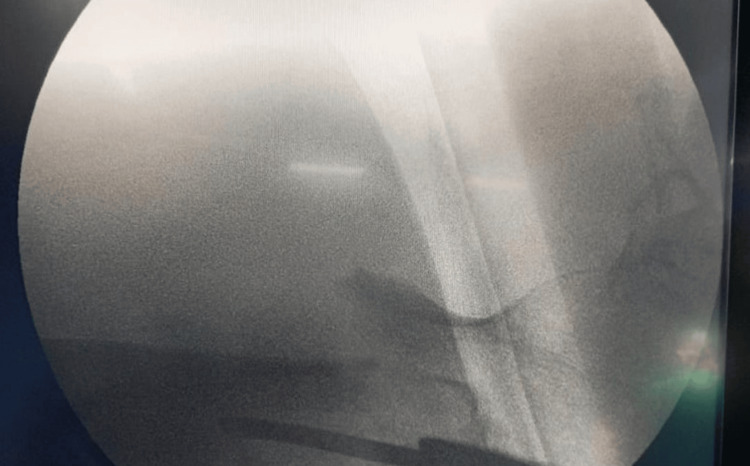
Lateral view head and neck displaced (irreducible fracture).

**Figure 8:**
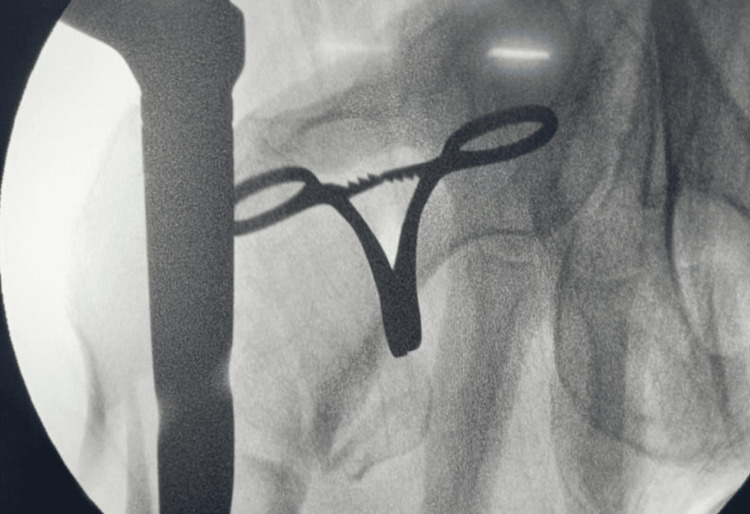
For reduction, a curved artery is used for alignment on the anterior-posterior image.

**Figure 9:**
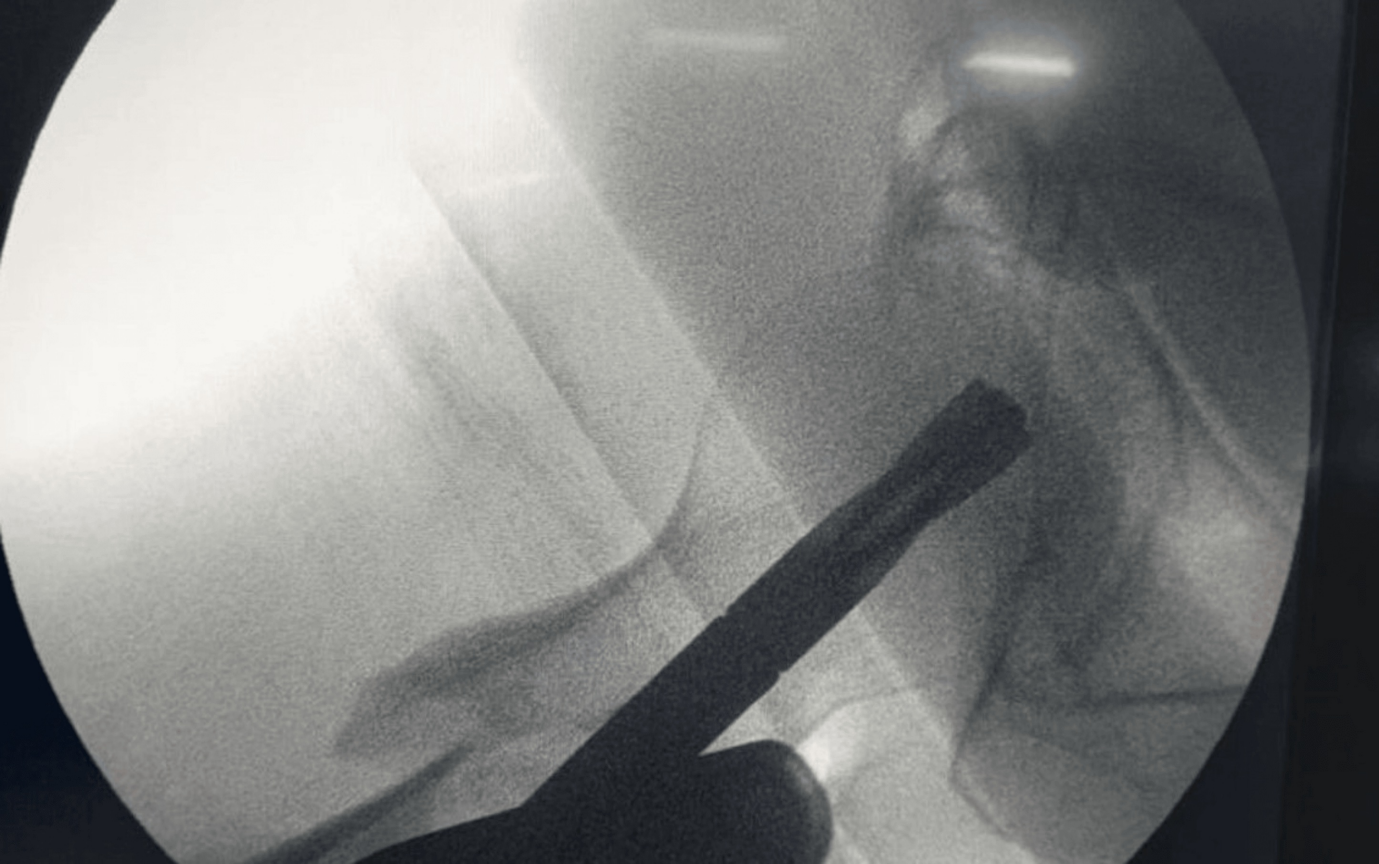
Lateral shows non-anatomical reduction with PFNA2 implant. PFNA2: proximal femoral nail anti-rotation

Less than 14% of fractures were difficult to classify as AO types [18,19]. Irreducible fractures were divided into various types, including interlocking of fragments and impacted fractures. These fractures were reduced by reducing traction, opening the fracture site, and using a clamp [20,21]. Typically, the fracture pattern consists of a flexed head and neck fragment, accompanied by a sagging distal fragment. We performed the reduction by manually pushing the femur up with a hammer, then applying an anterior spike to push it down for cortical contact (Figures 3-9) [22,23].

Some surgeons used a Steinmann pin anteriorly or an elevator to push the head and neck fragment while a hammer was used distally to lift [24].

Lateral wall fractures can cause collapse of the fracture and are not always present as irreducible intertrochanteric fractures as reported by some authors; prior cerclage wiring has been recommended [25,26].

Lateral wall fractures on coronal and sagittal planes have been considered irreducible fracture types; this is not always applicable. Some fractures reduce well on a traction table.

The point is for the choice of implant and use of reconstruction methods of the lateral wall to prevent collapse at fracture prematurely.

Limitations and strengths

This study has limitations due to a small sample size and the absence of CT scans. The strength of this study is that it is based solely on plain X-ray images of the pelvis. Surgeons may attempt to predict the irreducibility of fractures and assess the need for semi-open or open reduction methods to reduce operation time.

## Conclusions

Understanding fracture fragments that are irreducible may help surgeons refine reduction techniques, achieve anatomical alignment for fracture union, and reduce stress and operating time. The answers to the new questions are: not all unstable IT fractures are irreducible; irreducibility of the fracture is not always predictable or consistent; and the techniques that should be used, after lateral exposure where the helical blade is to be inserted, are to open it and use a bone hook; alternatively, use 2 Homann’s retractors (spikes) to reduce the fracture under direct vision care taken to prevent damage to femoral vessels. Steps of simple techniques are standard abduction and external rotation on the fracture table and then adduction and internal or neutral rotation on the table. If irreducible on C-arm imaging, the most commonly preferred is single incision posterior laterally over the trochanter, slitting the vastus lateralis and double spike techniques.
